# Asian Livestock Industry Leaders’ Perceptions of the Importance of, and Solutions for, Animal Welfare Issues

**DOI:** 10.3390/ani9060319

**Published:** 2019-06-05

**Authors:** Michelle Sinclair, Clive J.C. Phillips

**Affiliations:** Centre for Animal Welfare and Ethics, School of Veterinary Sciences, The University of Queensland, Brisbane, QLD 4343, Australia; c.phillips@uq.edu.au

**Keywords:** animal welfare, perceptions, China, attitudes, cultural anthrozoology

## Abstract

**Simple Summary:**

Livestock industry leaders are in a unique position to understand the challenges to the welfare of farm animals and to enact solutions that improve the lives of the animals they work with. Despite this, these important stakeholders are seldom consulted in this regard and their attitudes and opinions on animal welfare issues are largely unknown. This is particularly the case when considering livestock stakeholders in Asia and the previous lack of collaboration with international animal welfare advocates. To address this, focus group consultation sessions were organised across six countries; China, India, Malaysia, Thailand, Vietnam, and Bangladesh, aiming to better understand the positions of livestock industry leaders, and to consult them on potential initiatives to improve livestock welfare in their country. This study presents a ranking of animal welfare issues by importance as considered by livestock leaders, solutions and opportunities identified by the leaders, and suggests initiatives proposed in each country. This study aims to better inform international animal welfare strategies in order to facilitate the development of the most effective programs and initiatives.

**Abstract:**

The welfare of farm animals has been the focus of increasing international interest, however, the movement has had little engagement with livestock leaders who are, arguably, the stakeholders in the position most able to make decisions that impact on animal welfare at critical times. Previous studies have drawn attention to the need to engage in constructive collaborations with the livestock industry for the betterment of animal welfare, and to uncover mutual benefits for both stakeholders and proponents of animal welfare with which collaborations can be motivated. This study aimed to continue this need to understand leaders in livestock management, by consulting their opinions as to what constitutes the most critical animal welfare issues during farming and slaughter, and what they see as some of the solutions to begin addressing livestock welfare issues in their country. Seventeen focus group sessions were held with 139 leaders in livestock industries in six diverse countries in Asia, including China, India, Malaysia, Vietnam, Thailand, and Bangladesh. Leaders included government representatives, key academics in agriculture, and business managers and leaders within the domestic animal agriculture industries, as relevant to each country. After conducting thematic analysis and applying basic statistical measures, the findings suggest that solutions within the themes of education, training, and awareness are most valued. However, how each of these could be best addressed varied by country. The need for local research and local solutions also contributed to the most frequent opportunities, as did the requirement for prescriptive and consistent standards and expectations. A ranking of animal welfare issues is presented, as is a selection of suggested animal welfare initiatives resulting from the findings of this study.

## 1. Introduction

Animal welfare, particularly when considering livestock, is a social issue that involves more stakeholders than most: consumers, animal advocates, governments, the public, veterinarians, livestock industry and the animals themselves. Well understood in the realms of commercial marketing, political science, education, war, and corporate psychology, understanding the target audience, or relevant stakeholder, is vital to the success of campaigns [1,2,3,4] and a universal approach to stakeholders is unlikely to generate engagement. Although the principle of understanding audiences and stakeholders has not been widely applied to social enterprise, or more specifically to the purpose of improving animal welfare, it is directly applicable and is likely to result in the development of improved strategy and initiatives [5]. Engaging stakeholders in a relationship with a product, philosophy, or behaviour requires an understanding of who influences decisions to engage with a product, their needs and problems, what they are trying to achieve, and how they can be successful [6]. Identifying the right stakeholders to understand and engage with is, therefore, a fundamental component of this process. In the past, animal welfare science, along with the broader farm animal welfare movement, has been focussed almost entirely on the animals themselves, which is reasonable considering they are the main subject, and the stakeholders with the most to lose or gain. However, farm animals, and particularly those in intensive production systems, are often not in a position to improve their welfare, and are only rarely able to make choices that improve their quality of life. When considering the stakeholders with the greatest amount of choice over the details that have the greatest impact on the animals’ lives, and their welfare status, it is clear who the key actors in the livestock industry are: farmers, transporters, slaughtermen, and veterinarians. Livestock industry stakeholders could be considered the most important players in farm animal welfare for more reasons than being in an empowered position to directly implement animal welfare improvement. Apart from the animals themselves, no other stakeholder has as much to lose or gain from animal welfare initiatives, with stakeholders in many developing countries relying on livestock and subsistence farming for survival. In addition, no other stakeholder understands the intimate details of the livestock production business as well as they do, including the challenges and solutions to improving animal welfare. Contrary to the potential of this stakeholder group, livestock stakeholders in developing countries have seldom been the focus of animal welfare research or strategy. Some research has been undertaken on the understanding of attitudes and behaviour of livestock industry stakeholders in developed countries towards animal welfare, e.g., [6], but in the developing economies of Asia there are many different traditions and cultural influences, as well as market forces, which suggest that different attitudes and behaviour may prevail.

Groundwork research has been conducted to begin understanding the attitudes of livestock stakeholders [7,8,9], but only a few studies have been recently conducted with Asian stakeholders [10,11,12,13] despite the fact that the majority of livestock are produced there. The importance to finding mutual benefits (or ‘mutual gains’) with stakeholders has been presented in an international development context [14,15] and in the context of animal welfare [16]. Further to that, the nature of the perceived benefits for livestock holders, if they improve the welfare of the livestock, has also recently been explored [17]. Understanding livestock stakeholders’ perceptions of different animal welfare issues, and stakeholder-initiated solutions or opportunities to improve animal welfare, has not yet been researched, particularly not in the context of Asian countries.

This study aims to draw on the skills and experience of livestock leaders across six culturally diverse Asian nations with economically important livestock industries to begin addressing this gap. Incorporating stakeholder-identified opportunities to improve farm animal welfare into international initiatives is more likely to be supported by these stakeholders. Furthermore, this study aims to provide an improved understanding of the stakeholders tasked with making the most critical choices on behalf of the animals and their welfare.

## 2. Materials and Methods

The study was granted human ethics approval by the University of Queensland Ethics Committee, approval number: 2017000628. Three focus group sessions were held in each of five countries: India, Vietnam, Thailand, China, and Bangladesh, and two were held in Malaysia because of the smaller population, relatively. There were a total of 17 focus groups and 139 livestock leaders (Table 1). Locations were dispersed in each country (i.e., south, north, central, capital, and regional), in an effort to capture potentially varied sentiments between different geographic regions. Participants (henceforth referred to as stakeholders) were invited through country-based collaborators utilising the following selection criteria: they had to be leaders in the agricultural sector, senior within a private organisation or agriculture government department (with a maximum of five government vets), and considered to have the ability to implement change in private businesses. The majority of stakeholders were private industry leaders, such as pig or poultry slaughterhouse or production managers or owners (Table 2). In some focus groups, certain participants were known to each other as professional colleagues.

Although plans were made for five to seven stakeholders in each session, the actual number of stakeholders present on the day varied from three to 14, due to cancellations at the lower extreme and heightened interest at the other. Sessions were scheduled for 3.5 h, but those with more stakeholders often ran past in order to allow all stakeholders adequate opportunity to contribute.

The lead researcher (MS), with the assistance of a research assistant, facilitated all groups in a semi structured format with consistent base questions (see Appendix A), followed by prompting stakeholders for further discussion around animal welfare issues relevant to them, which suggested strategies, solutions and opportunities to improve animal welfare in their country. In addition to this discussion, two activities were conducted with stakeholders that are presented here: a group ranking of what stakeholders saw to be the most important livestock welfare issues, and an individual rating of the perceived willingness to improve stock handling skills to be calmer and gentler with livestock animals, in the context of front line animal staff within the livestock industries. Within the group activity, 12 cards were presented with major welfare concerns in livestock production, written in both English and the local language, translated by the local academic collaborator (simplified Chinese in China, Vietnamese in Vietnam, Thai in Thailand, Bangladeshi in Bangladesh, Hindi in India, and Bahas in Malaysia). To avoid presenting misleading data, linguistics and tone are not reported, as most data was translated, abbreviated, and summarised through verbal translators during the sessions, from six different languages to English.

Stakeholders were asked to reach a consensus as a team and place the cards in order of importance, from the most concerning animal welfare issue, to the least. This was first completed in the context of animal farming, and second in the context of animal slaughter. Stakeholders were encouraged to discuss their thoughts, and present their rank order back to the facilitator and research assistant when they had agreed on an order. The research assistant advised the groups by answering questions about the meanings of cards if they were not readily understood. In each session, the stakeholders were advised to consider the issues not in relation to the current issues on their respective farms, rather, that in the case that all of the issues were present, which issues were the most important, ranked from most to least important.

The task took between 10–30 min in each session, with some groups choosing not to distinguish between farming and slaughter, and to present one list. The sessions ended with a summary of the major points presented by the lead researcher (MS), in order to achieve agreement with all stakeholders. Stakeholders were not paid for their participation, were advised that the session was voluntary, and that all data would be de-identified.

The remainder of the session content focussed on the benefits to improving welfare, willingness to embrace pre-slaughter stunning, and, pursuant to our earlier surveys, achieving a better understanding of the motivators to seek to improve animal welfare [10,11,12,13,16,17].

Dialogue was voice recorded during the sessions and additional field notes were taken by the research assistant. Both datasets were used to create abridged transcripts of each session. As participation was facilitated by verbal translation during the sessions in most instances (except for some Malaysian, Thai, Indian, and Bangladeshi participants who spoke English), verbatim transcripts were not possible. However, post hoc transcripts were uploaded into NVivo 11.4.3 (QSR International, Melbourne, Australia) software for Mac for analysis (henceforth referred to as NVivo). The same lead researcher that conducted the focus groups also coded all themes (recurring concepts or ideas [18]) and conducted the analysis. On completion of each session participants received a small gift from Australia (such as pins, magnets, and small koalas) as a token of appreciation for participating in Australian research.

### Analysis

For the data collected in the animal welfare issues group activity, the ranking of issues per location is presented as it was collected, and the percentage of total solutions that each solution comprised was calculated and is presented graphically. Thematic analysis was utilised to obtain a deeper understanding of the solutions and specific opportunities, and to investigate the most important solutions to stakeholders. Data contained in the session summary and relating to solutions and opportunities was compiled into 19 solution themes and presented as they appeared in each location. The percentage of total solutions was calculated for each individual solution. The most significant solution theme was then further analysed for specific sub-themes and what proportion of this solution theme each constituted. Once all data relating to solutions or opportunities to improve animal welfare had been coded as a theme, the data was manually inspected for quotes that best illustrated this data. At the completion of analysis and coding of themes and sub-themes, no new themes emerged from the data, suggesting data saturation.

Due to possible translation nuances, rather than focussing on word usage, more attention was paid to careful analysis of the key themes, the frequency of their appearance across countries, and the general context and interpretation of their meanings. However, word frequency functions in NVivo were still utilised in the identification of sub-themes and to ensure data saturation.

For the data collected in relation to rating the willingness of stakeholders to engage in calm and gentle animal handling, responses were counted for each location, and presented as a mean and median. Data relating to these scores were coded accordingly and are summarised in the results.

## 3. Results

### 3.1. Animal Welfare Issues

**Figure 1:**
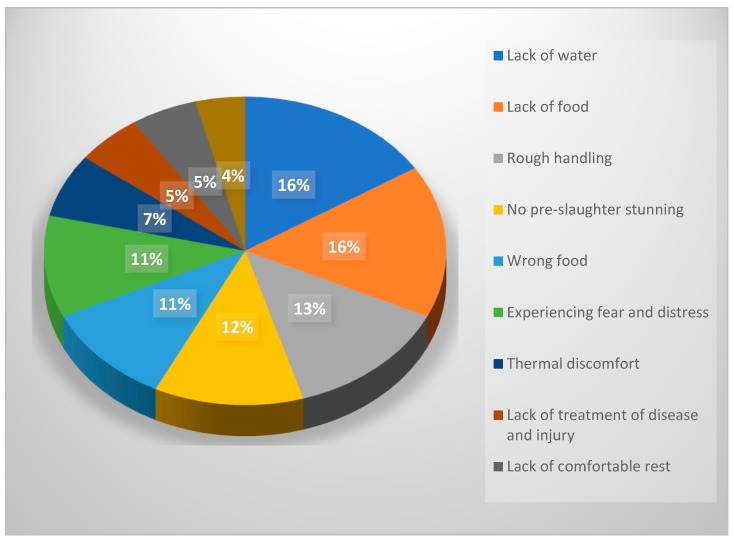
Percentage of total solutions by the focus groups citing each animal welfare issue in farming and slaughter rated in the top three ‘most important’. Note: The 12th issue, ‘boredom’, did not appear amongst the top rated animal welfare concerns in any of the sessions, therefore, it does not appear here.

In a farming context, most stakeholders placed the lack of food and water at the top of the list as the most serious animal welfare issues (Table 3; Figure 1). Lack of pre-slaughter stunning, coupled with experiencing fear and distress and rough handling, were rated as most important across most countries when considering a slaughter context, except in Bangladesh where the lack of pre-slaughter stunning was rated amongst the lowest animal welfare concerns during slaughter, and one session in Malaysia in which it was placed in the middle of the list.

In those sessions in which the stakeholders chose to craft one list to cover both farming and slaughter contexts, consideration was frequently given to the lack of pre-slaughter stunning in Vietnam and India, and to thermal discomfort in Thailand. Boredom consistently ranked the lowest of animal welfare considerations in all sessions, both in the farming and slaughter context, except in one session in China in which rough handling and lack of shelter were rated as being of lower in importance in a slaughter context, and in one session in Malaysia in which, again, rough handling rated lower than boredom in a farming context. In the slaughter context boredom was rated at a low level, however, it was rated of greater importance than food and treatment for disease in a couple of sessions, presumably due to the withholding of these provisions prior to slaughter. Lack of stunning at slaughter was mostly rated the least important consideration in Bangladesh, interchangeably with boredom.

Most other animal welfare issues were rated with varied importance, with no significant consistency by country, except that space to express normal behaviour, thermal discomfort, and rough handling considerations rated consistently high in most countries when considering farming, after food and water, with the exception of Bangladesh, which had less consistency in the rating of these issues.

### 3.2. Stakeholder Initiated Solutions or Approaches

**Figure 2:**
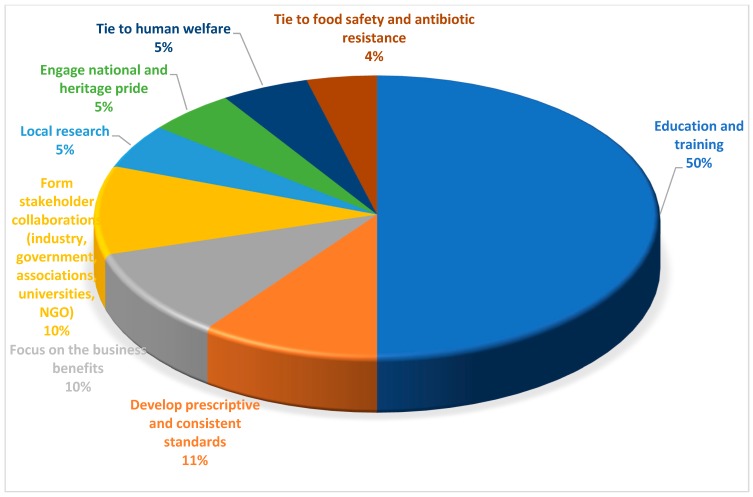
The top 12 stakeholder suggested solutions and opportunities to improving animal welfare (% frequency with which each of the top 12 solutions was represented, across all countries).

**Figure 3:**
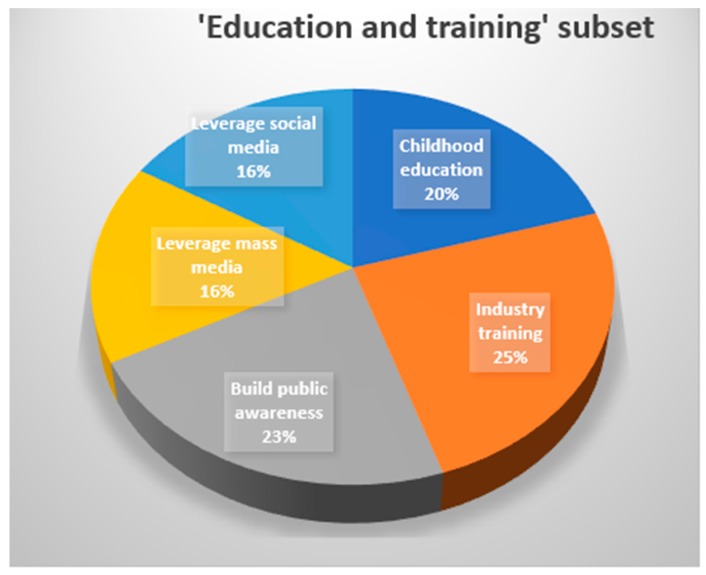
Visual representation of the % frequency of subsets within the ‘Education and Training’ solution, across all countries.

The primary themes underpinning the solutions presented by stakeholders are summarised in Table 4 and Figure 2. The most frequent were education of children, industry training, awareness of the general public, the need for prescriptive standards, and the need for local research and local solutions. Although some of the themes were consistent across the countries, the way in which each was suggested to be conducted and their content varied considerably. For this reason, and for practicability of application, the qualitative detail of these themes are considered and discussed by country, illustrated with direct quotes from the stakeholders. The subthemes for education and training were, from most to least important, industry training, building public awareness, childhood education, and leveraging social and mass media (Figure 3).

#### 3.2.1. China

The solutions presented with the most frequency in China centred on a practical and pragmatic approach. Firstly, the creation of industry standards that are prescriptive by nature, locally relevant to China, and based on scientific measurements (Table 4). “During the conference of the last October in Hangzhou the wise Minister of China Agriculture expressed (the view) that we cannot have animal welfare standards that are not realistic in the Chinese situation, they must be based on our own situation, not based on other countries” <BJ>. “Animal welfare is never ending, conceptually, so we need specific standards and guidance on how to meet them…because you cannot meet the requirements of a concept” <BJ>.

The second solution presented in all sessions in China was the need to present clear information on the business benefits for improving animal welfare. “Companies want to make money, so when animal welfare can improve their benefits they will incorporate that notion” <BJ>.”Is there any specific data to prove a positive connection between animal welfare and efficiency of economic benefits to company? If we have such data it will become much easier to promote the concept” <GZ>. “We need to communicate that animal welfare can improve their productivity” <ZZ>.

Five other solutions were repeatedly presented with emphasis in China, though not in every session. One of these focussed on building a body of local research that is Chinese led, and specifically relevant to the Chinese industry and conditions. “We have all the foreign countries pressing us to go forward on animal welfare and that will make us confused… the foreign countries have advanced animal welfare practices and they are pushing us to go like them but we don’t have the fundamentals <BJ>.

“We need to encourage the Chinese academics to promote the research, first the scientific knowledge. It is easier to trust Chinese science conducted in a Chinese environment” <BJ>.

Collaboration between stakeholder groups, in particular the importance of working with Chinese government, was raised in each session. “The government is very important to the Chinese people, for example, if government media says ok we need a green environment, it will engage the common people” <ZZ>. “The government need(s) to consider that if we have better animal welfare we may have more advantages in international trading or leading positions ahead of other countries” <BJ>. “If the government focusses on this people will follow, but if not, they won’t do it (animal welfare improvement)” <GZ>.

The last three of these could be grouped together as focussed on raising awareness of animal welfare amongst the general public, and lifting the profile of animal welfare both in social media, and mass media. “First we should rely on the internet and second is that we can use documentaries to promote (animal welfare, including) social media such as Weibo and applications with live videos such as Huajiao” <BJ>.

#### 3.2.2. India

Education focussed on children was a primary solution to improving animal welfare, according to Indian stakeholders. “Everyone in the general public, right from school children age should be educated… not only about dogs, all animals should be treated well and it should be part of the curriculum. It should be made mandatory if they’re involved with animals or not, because they will come across animals” <BA>.

For both children and adults alike, a pride in the ‘goodness’ of Indian heritage; the history of belief in the sacred nature of life and the holiness of animals could be used as a platform to convey messages of respect and empathy that would underpin behaviours that seek to advocate for the welfare of animals. “Indians are the people who worship animals, so taking care of their welfare is equal to taking care of God, but most people are not aware of that…so you can direct things in that angle.” <TR>. “Consider gods of Hindus, every god is related to some animal, and we had a great culture of worshipping these animals; welfare is taking care of needs, people will see that as caring for gods” <TR>.

Stories traditional to Indian heritage could be an important tool to assist in this purpose. “When we were kids there was a set of tales all about how animals and humans co-exist and how humans learn lessons from animals. It’s sad that in today in schools those tales are not taught to children. We are copying the West and taking your stories and lessons, but these short stories are beautiful; how much we interact and benefit from symbiotic relationships…this could be done in schools” <KO>. “Being kind and compassionate to animals is there in our history, mythology and stories, we have grown up with it. However, over time we are living in urban pockets with shopping malls and we have lost the connection with animals and that they too feel pain and suffer; we have to start re-educating our children and society.” <KO>.

Utilising social media to build awareness around animal welfare issues, and to elicit empathy and concern was also raised in each session in India. This included the use of Indian celebrities (specifically Bollywood stars and cricketers), which was deemed to be an important medium to elicit interest. “It is very popular in India to use celebrities; for example in India, cricket is religion…we had an Indian cricket captain speak on TV about vaccination for rabies...involving celebrities has a very good impact” <BA>. “Youtube and WhatsApp are popular platforms” <TR>.

Other solutions presented in most of the sessions in India included a need to understand the complex local issues through research, including all stakeholders impacted by potential solutions, which could then result in a holistic ‘roadmap’ to solving the issue. “Basically, India is very vast country with so many kinds of issues and we don’t have a program like a road map to achieve what we can do. It’s a little like traffic, you people would be shocked, however, we cannot have traffic rules like those which exist in Australia…What applies to your country may not apply to our country, so we need practical local road maps” <BA>. 

One Welfare, the need to focus on human welfare and livelihood in solving animal welfare issues, was reiterated with wide agreement in most of the sessions. “So where human welfare is a problem how do you think about animal welfare? They both need to be addressed at the same time” <BA>. “Unlike in other countries you can’t see animals as a separate entity to the people; animals form an important and integral part of our livelihood” <TR>.

Where this approach to solutions had been attempted, stakeholders shared success stories. “We stopped bear dancing in India, we did not just say stop, we re-employed them and gave them new professions…same with snake charmers, snakes are confiscated and we give alternate professions. So we should not only approach the animal welfare, but ensure the welfare of humans is not compromised…especially in India where 70% of population goes to bed without a meal” <BA>. This notion fed back into the need to conduct comprehensive research with stakeholders before putting initiatives in place.

Lastly, on the implementation of law in India, it was felt that initiatives that increase the implementation and monitoring of the existing laws may aid solutions, however, it was believed to be a highly political landscape, and therefore, a complicated route. “India has the most advanced animal welfare laws, we are the only country where animal welfare is mentioned in the constitution, but for so many political reasons it lacks implementation. One reason is that every slaughterhouse, barring a few, is owned by Muslims and it is a politically sensitive issue (with the government predominated by Hindus). If you go with police and stop something at the slaughterhouse there will be riot, there will fighting and there will be dying…so no government will dare take on issue, they have bigger problems. In some places the slaughterhouse is locked from the inside; what happens inside nobody knows…nobody can do anything as (it is) politically sensitive” <KO>.

#### 3.2.3. Malaysia

The need to educate children to plan for a society in which animal welfare notions are received with more openness was presented as a priority in all of the Malaysian sessions. The nature of education in Malaysia, however, was thought to benefit from a more technical focus on the animals themselves, and the basics of agriculture. “Children need to be taught about agriculture, where eggs and milk come from…doctors, accountants and lawyers…no one has any idea about where anything comes from. Then the next step is to talk about animal welfare…it would be much easier to talk about animal welfare when they have a base understanding” <NS>. “Increasing personal value should start at school with children, they need to realise that animals are creatures of God, created by God and we should appreciate them” <KL>.

Building public awareness around what animal welfare is, the links to product quality and the benefits relating to animal welfare were considered beneficial. “People need to be educated on animal wellness and welfare and also food security. Providing a safe and quality product can all be related to in animal welfare education” <KL>

The Department of Veterinary Services (DVS) within the Malaysian Government were thought to be in the best position to share these awareness messages, as the authority that ensures the requirements of both the religious body in Malaysia, Jakim are met (such as the production of halal), along with those of the government, and the animals). “Education should be through DVS and it should be standardised, otherwise everyone has their own thoughts” <NS>. “DVS are now organising seminars, talks and one-on-one sessions, support is free…get DVS to advise on animal welfare and they can dispense the advice to farmers” <NS>.

Collaborations between DVS and other important bodies, such as Jakim and universities, with livestock industries and NGO bodies, were seen as the only way to progress animal welfare issues with wide reaching impact in Malaysia. “Religious authorities know about the importance of animal welfare but they don’t know what happens on farms so they don’t know how to improve it; it is a matter of education between religious authorities and DVS as technical advisors” <NS>.

The last solution that was presented by stakeholders in all of the sessions was that of presenting livestock business managers with the financial benefits for improving animal welfare. “I agree, education is a must, and without education we don’t understand the importance of animal welfare…but for the businessman profit is a must; if you can show them by doing this (improving welfare) you will improve your profit, the boss will tell them do it now, not tomorrow, now!” <NS>.

#### 3.2.4. Thailand

Reliance on government offering industry training to improve animal welfare was one of the two stakeholder solutions that were presented in all of the sessions in Thailand.

“Previously we just have been told to do animal welfare, not how to do it, so that detailed information would be good...for example, instead of telling us that we need enrichment for the animals, tell us what it is, and how we can make it” <BK>. “In general I think Department of Livestock (DOD) are in the best position to look after this, with officers not only in the central areas but also districts and regional areas” <BK>.

In line with this, the other solution presented in all Thai sessions was creating a collaborative animal welfare network that comprised the Thai Department of Livestock (DOD), livestock industry leaders, and academics. “Department of livestock, local agricultural officers and universities or colleges should work together. The DOD should be the main organisation but the officers need to deal with the farmers after the university or college build the officers knowledge” <CM> “I think everyone works in a silo at the moment. Everyone aims to improve animal welfare, but the scientists only research and the businesses only focus on business. Who will lead us to bring our groups and stakeholders in line in animal welfare?” <BK>.

The notion of peer knowledge sharing between livestock stakeholders, and between knowledgeable farmers, was presented in most of the sessions in Thailand, as was the potential utility of livestock associations in the facilitation of this process. “We have success when we see another farm succeed. In smaller farms you may try something but have obstacles, but once other farms have the same experience and find success then it is easier to learn from others and implement it” <BK>.

“(People on) small farms in villages are friends anyway and they might form an organisation or association to exchange ideas. So they might come and share and influence others. They have organisations of pig farmers, chicken farmers, and beef association and so on, so they can organise for members to share knowledge” <BK>. “The associations communicate with each other on things like Facebook and (on-)Line” <BK>.

A pride in the existing kindness and empathy inherent in Thai culture was evident in Thai sessions, and for this reason it was thought that training initiatives could be more usefully focussed on how to implement better animal welfare, rather than why to do this. “Thai people are kind, I think our personal value may be related to our Buddhism; as a Buddhist the first rule is be kind to animals, to the living things” <BK>. “They think that as farmers they don’t know what animal welfare is, in terms of definition or phrase, but when we take care of animals we want our animals to be comfortable, to be healthy…Farmers will do all kind of things to make sure their animals are well, things that might collectively be focussed on animal welfare…some things they do by good intention though (they) may not be good for animal welfare” <BK>.

Building the profile of animal welfare through social media was also presented in most Thai sessions, with messages in line with the aforementioned solutions; an emphasis on Thai cultural kindness by the general public, and information on successful practical methods of improving animal welfare from ‘knowledgeable farmers’. “Cooperation between friends and peers will help with the ability to improve animal welfare, farmer to farmer. In Thailand right now, especially in rural and regional areas, there are people who will hold a lot of knowledge and are very successful, a lot of farmers from everywhere will come and learn from them” <CM>.

Lastly, although the need to create clear and consistent animal welfare standards as a solution was only presented as a solution in one session, it was presented with great emphasis from stakeholders in that session. Confusion and frustration existed at one of the largest animal production companies in Thailand, within which an animal welfare agenda was active, with evolving policy aimed at improving animal welfare standards for export market reasons. “There are many different kinds of law and different countries will be slightly different…so which law we should follow? We are in Thailand, but once we export do we follow the Thai law? We have to focus on country law and our customer, the European Union (EU) may say this stocking density is acceptable but the buying company may disagree. We can say this is EU law and we comply but they say no we must meet our company law” <BK>.

#### 3.2.5. Vietnam

Providing industry training on animal welfare was raised as an important solution to improving livestock animal welfare in all of the Vietnamese sessions. “Training is first priority for stakeholders who directly work with animals” <HCM>.

The leader of one of the largest beef importers in Vietnam attested to the likelihood of success of practical skill-based stakeholder training programs. “From our company’s perspective, some years ago I knew nothing about animal welfare and some other people and I were trained by an Australian export company. I became aware of the importance of animal welfare, then I read and got more knowledge and I trained my staff. Our industry partner company initiated the training and it was run by MLA (Meat and Livestock Australia)” <HCM>.

The other solution raised in all of the Vietnamese sessions was again that of childhood education. In this instance, a concern for animals was believed to be currently lacking, and could be fostered in children, eventually increasing the uptake of industry training for livestock stakeholders. “All children should be educated about animal welfare for basic knowledge, and then as they grow up animal welfare grows too. The problem now is not a lack of care, just that the knowledge is poor. Education for children is for the future, while specific training for stakeholders is for short term” <HA>.

Additionally, raised in most, but not all, sessions in Vietnam was the need to raise public awareness that animal welfare is a consideration, which feeds into the need for childhood education, and also to focus awareness of the livestock stakeholder leaders on the financial benefits of improving animal welfare. “Companies are big business, so if they know that if you apply good methods it will increase profits they can invest, as they have the money and systems to do it, and the training” <HCM>.

#### 3.2.6. Bangladesh

In Bangladesh, the need to focus solutions on human welfare and livelihood was presented in every session, with wide agreement from stakeholders. “There are more major issues than animal welfare here…human rights in Bangladesh is hard, so how do you think about animal welfare?” <MS>. “If we want to improve animal welfare we have to think of farmer welfare also as they are closely related. Most animals come from family production, about 60%, they are poor and their livelihood depends on farming” <SA>. “No farmer welfare, no animal welfare” <SA>.

Further to this, all stakeholder sessions proposed that the creation of prescriptive and consistent standards for improving livestock welfare would offer a solution to improving animal welfare, as the knowledge levels are basic and it is not known how to improve animal welfare. “First the government must build guidelines” <SA>.

In place of rigorous scientific knowledge around animal welfare, laypeople (defined by stakeholders as those treating animals with no professional training or professional degree) have become prevalent in Bangladesh, causing problems for the health and wellbeing of the animals they treat.

Following this, offering industry stakeholder training was the last solution presented in all Bangladeshi sessions, with the target audience to include government representatives. “First we need good training materials and then we should have master trainers…then they will make another group of master trainers, and this will be continued. Nowadays everyone has smart phone and wants to play with it, they like it. Farmers too…so farmers can have access to those materials and they can see and understand easily. By looking at that material again and again maybe this will come into their heart. Facebook is also very popular here” <DH>. “The big stakeholder is DLS (Department of Livestock Services). DLS personnel should be trained properly (to be) number one, then they can train the farmers up. The training should be a continuous process” <DH>.

Additionally, raised in most of the sessions was the need to build public awareness to support the profile of animal welfare, primarily through traditional mass media, specifically television and radio. “We need to develop awareness…we have a TV cartoon in Bangladesh that plays an important role in women’s empowerment and the basic needs of human health, a similar attractive TV program for children on animal welfare could help make a sustainable change for the nation” <DH>. “Currently our government has an advertisement about polio vaccinations, we need something similar for animal welfare” <SA>.

Further to the solutions that focus on animal welfare as a complementary cause, rather than a stand-alone endeavour, stakeholders in most of the sessions in Bangladesh emphasised that food safety and antimicrobial resistance were more important issues in Bangladesh, but had the ability to be widened to include animal welfare. “Two issues are more important, one is food safety and the other is antibiotic resistance…If you want animal welfare to be popular you must tie animal welfare to these” <SA>. “One example from two years back, our society was not aware of antimicrobial resistance, but in the last two years in collaboration with DLS and under a One Health approach they raised awareness, and nowadays people are aware about AB resistance…so animal welfare should start now” <DH>.

In relation to themes, stakeholders in most sessions proposed that solutions should be focussed on the human benefits for improving the welfare of animals, including improved productivity and profit. “First we need motivation…if I understand what benefits I might get if I follow an animal welfare path and understand the advantages to our economy and to public health then we can easily accept (it)” <SA>.

Citing that animal welfare is a brand-new consideration in Bangladesh, one stakeholder made a proposal to other participant stakeholders on conclusion of the session; “maybe we could make a network of people interested in animal welfare. Maybe we are ten people and after six months 50% will drop out but we have a platform to extend our views and share new idea” <DH>.

### 3.3. Improving Animal Handling Skills

In response to the question, ‘on a scale from 1–10 how willing do you think people handling the animals would be to improve their stockperson skills to be calmer and gentler with the animals’ stakeholders provided a rating (Table 5). After providing ratings, stakeholders were asked for suggestions to improve the willingness of stock handlers to improve their skills to be more gentle and calmer with the animals. The most common response in all countries was that catchers and movers needed more time, as they are often working quickly to either meet demands, or to get paid for their job and move on, mostly being contractors or casuals recruited from a foreign contingent.

Suggested solutions included incentivising the absence of injury to animals and good carcass quality, hiring a sustainable workforce who are then trained appropriately, hiring workers with the right personal characteristics to be gentle, structuring the pay differently, offering staff rewards for careful catching, caring for the handlers to ensure they are not tired and are well rested so as to not let their work slip, looking into machinery to replace workers, investing in better tools for the workers to use, better advertising the benefits of gentle handling, teaching handlers about animal behaviour, reviewing management processes to ensure fewer animals arrive at slaughterhouses at the same time, monitoring and supervising the process both in person and using CCTV, issuing penalties for rough stockmanship, and researching and counteracting the effects of desensitising to suffering.

## 4. Discussion

### 4.1. Issues

In regards to the ranking of issues, it is noted that most stakeholder groups presented them in order of importance in line with Maslow’s Hierarchy of Needs, a pertinent human model of psychological motivations [19]. That is, basic physiological needs of food and water were presented as the most important issues in most situations, as they are for humans. Anthropomorphism, the practice of projecting human feelings onto other species, is often the subject of caution, as it is not based on a scientific knowledge of the animals and their needs [20]. However, in many instances it helps humans to relate to, and empathise with, other species [21,22], and in the context of this study could be useful. On multiple occasions in different countries, stakeholders shared their experiences of training farm staff and slaughter staff to describe how animals may feel in the environment, too hot or too cold, for example, and they believed that it is important to be aware that the animals may have similar feelings.

However, caution is still advisable as culture is obviously influencing the perceived impact that certain animal welfare issues, such as the lack of stunning at slaughter, have on animals. Although the lack of stunning at slaughter was rated with high importance overall, immediately after lack of food, lack of water, and rough handling, in some countries (Bangladesh and Malaysia), a lack of pre-slaughter stunning was not seen as a significant concern for the animals and their welfare. This perception is likely to be based on religious belief, with most Bangladeshis adhering to a traditional sect of Islam that believes that the preparation of halal food, i.e., that which is permissible for Muslims to eat, needs to exclude pre-slaughter stunning, with the practice seldom conducted during slaughter [23]. Although animal welfare science demonstrates that pre-slaughter stunning improves animal welfare [24,25,26], the belief that it is not acceptable seems to be transferred to the perceived experience of the animals regardless.

Boredom was consistently rated as the lowest animal welfare concern, with some stakeholder groups purposefully leaving it out of the activity, confused as to why it was included as an animal welfare issue, and in some cases, met with scepticism. This may indicate a lack of understanding of the concept of mental welfare in animals, as well as animal behaviour and enrichment.

This activity was a valuable insight into the perception of stakeholders in different countries, and provided indications as to which animal welfare issues are likely to be more easily acknowledged and accepted by industry stakeholders in each region.

### 4.2. Solutions

The solutions and opportunities presented for addressing animal welfare expectedly varied between countries, in line with findings of previous studies with students and the general public [27,28,29]. However, the key sentiments of education, training, and awareness remained universal.

Despite this, the level of emphasis on education, training, and awareness and how best to conduct initiatives differed between countries. Solutions in both India and Thailand were community-focussed, reflecting the interconnectedness of animal welfare to people’s livelihood in India, and solutions based on knowledge sharing which, in Thailand, was proposed to be through industry association groups and farmer peer groups. Thailand is a community-based society, ranking very high on the Hofstedes cultural dimension of ‘collectivism’ (‘we’ rather than ‘I’), which may, in part, explain this finding [30]. It also reflects previous survey findings in Thailand that show that livestock stakeholders are more encouraged to improve animal welfare when it is important to their peers [10]. A focus on solutions that leverage a heritage of kindness in Thailand, and respect and reverence in India is understandable in light of the Buddhist and Hindu beliefs surrounding empathy and animal gods, respectively, and the large proportion of Buddhists in Thailand (93.2%) and Hindus in India (80.5%) [31,32].

Stakeholders in India, like those in Bangladesh, emphasised the importance of human welfare, and that animal welfare improvement should not be addressed in isolation, and where it is it will not be successful or sustainable. This concept of ‘One Welfare’ recognises the interconnectedness of animal welfare, environmental sustainability, and human welfare and advocates holistic solutions based on a knowledge of these interactions [33]. Of the countries investigated, India and Bangladesh have the highest rates of poverty, at 21.2% and 14.8% of the population, respectively, and the lowest income per capita, which may explain the emphasis on human welfare and livelihood in these areas as compared to the other countries [34].

While mentioned in other countries, such as China, the suggestion to tie animal welfare to other issues considered as public safety, such as food safety and biosecurity, was particularly prevalent in Bangladesh. The relative scarcity of both human and animal healthcare in Bangladesh probably explains this [35].

In China, unlike the other countries, education and industry training was not presented as a solution to improving animal welfare. With a strong focus on tertiary education within China [34] this finding was unexpected, however, it is also explicable for this very reason. According to data collected in 2014, expected years of education in the Chinese population were higher than those in the populations of other investigated countries [36]. Quotes from the Chinese stakeholders suggested that it was believed that the education and training levels were sufficient, but that there was a lack of prescriptive scientifically-supported standards or guidelines that were locally relevant to China.

While the presence and impact of animal welfare law was not the focus of this study, given it was not a solution stakeholders felt they had control over, it was intermittently thematic in these sessions, particularly as its absence presented an impediment to solutions. Animal welfare law is at different stages in the various countries of this study: China has no national farm animal welfare legislation, and it has been argued that these would be better implemented at a local level anyway [37]. In contrast to this India has had animal welfare legislation for almost 100 years, which illegitimises many cruel acts relevant to farm animals and has been updated several times, with the support of the Animal Welfare Board of India [38]. Bangladesh still has the animal welfare legislation enacted in colonial days but, recently, it has been utilised with greater frequency [39]. Malaysia, Thailand, and Vietnam have just introduced new animal welfare legislation, in 2015, 2014, and 2018, respectively, which has similar regulatory control of cruel acts to farm animals as India [40,41,42].

Indian stakeholders in every session presented with pride the fact that India had drafted more animal welfare laws than any other country, however, this followed with exasperation that the laws were not practicable or implementable in most cases. Likewise, in one session in Thailand, stakeholders demonstrated frustration with the lack of clarity around which animal welfare laws and standards they should follow, compounded when they are seeking to export to companies in western countries. With that, the contrast between environmental conditions in Thailand and the importing countries in Europe was emphasized. By way of example, one stakeholder shared that regulation for importers in the transport of chickens was that they were not to be sprayed with water, for welfare reasons. However, spraying the birds with water in the humid climate of Thailand was sometimes conducted for the very same reason: animal welfare. Likewise, electrical stunning standards set in Europe to ensure an effective stun of poultry is set for the welfare of the birds, however, when replicated in Thailand on smaller poultry species the birds are often killed by the same process. This might suggest a need for regional flexibility in international standards, with outcomes based on the animal and its welfare. A shift from a universal approach to standards, to respectful international relations and regional tailoring would minimise confusion, frustration, and maximise uptake of standards. Stakeholders in all countries stated that they would be willing to adhere to standards for improved animal welfare if they were practicable, based on science, and locally tailored. This then suggested a need for local collaborative research in each region to ascertain what the specific animal welfare issues might be, who the key stakeholders might be, who is in a position to influence the welfare of the animals, and what might enable them to do that. The result would be an understanding of who to approach, with what messages, and what support to approach them with. This was a focus particularly in India, where the sociopolitical environment is complex, chaotic, and sometimes contradictory.

To summarise the sentiments of education, training, and awareness that was a prominent theme throughout this study, in all countries children need to be educated on the value of the animal, what they are, and why welfare is important, and the general public need to be informed on farming practices so that, as consumers, they will pay more for higher-welfare products. Additionally, the livestock industry needs educating on the potential benefits of addressing animal welfare and how to implement it, and government officers, lawmakers and law enforcers need education to understand what animal welfare is and, often, the detail of the standards for improving animal welfare in their country. Finally, animal welfare scientists and advocates need education in the realities of the livestock business. To this purpose, multistakeholder collaborations could underpin major progress in animal welfare around the world.

### 4.3. Application

Below are listed potential opportunities to improve international animal welfare strategies and tailor them by region, derived from the findings of this study (Table 6).

While the findings of this study may be applied to improve animal welfare initiatives, it is important to note that solutions shared by the stakeholders are indicative of their perceptions and each suggested solution needs to be holistically assessed, considering all relevant stakeholders, and the socio-economic and political landscape in each country as to their suitability. Additional societal- and market-driven solutions also exist to improving animal welfare, to which an integrated global market could further assist. A limitation to this study exists in the variability of group size, from three stakeholders, to 17 stakeholders in the largest. This could have impacted the group dynamic, and therefore, the responses that were shared. While groups that numbered less than the intended minimum size of five (four in Trivandrum, India and three in Khon Kaen, Thailand) constituted a useful mix of government and industry stakeholders (two government/two industry, and one government/two industry, respectively), some limitations in stakeholder role diversity exist. One important example includes the lack of private veterinarians in the Chinese contingent of participants.

## 5. Conclusions

Livestock industry stakeholders have a good understanding of their industries and are in an ideal position to identify problems, find opportunities, and to enact solutions regarding animal welfare. Therefore, engaging them in farm animal welfare initiatives should, preferably, be the first stage of any strategic plan to improve animal welfare. This study suggested that solutions centred around education, training, and awareness were likely to have a great impact in most countries, however, the precise details of best practice implementation of these solutions varies with region and socioeconomic and political landscape.

The need for local research to develop local solutions, and an improved understanding of animal welfare supported by clear and consistent prescriptive standards was also evident. Through this animal welfare assessment focus can be on relevant outcomes, rather than utilizing a common set of criteria that may have weak links to animal outcomes. Bearing this in mind, optimal strategies are presented for each country.

The findings of this study can be applied to increase the background understanding of those who wish to be animal welfare proponents, to advise on the creation of an informed animal welfare strategy that is most likely to see success.

## Figures and Tables

**Table 1:** Location of focus groups and abbreviation codes used in quote citations.

Country	City/Town	Abbreviation Code	Number of Participants
**Vietnam**	Hanoi	HN	7
	Ban Me Thout	BT	5
	Ho Chi Minh City	HCM	8
**Malaysia**	Negeri Sembilan	NS	6
	Kuala Lumpur Selangor	KL	13
**Thailand**	Bangkok	BK	10
	Khon Kaen	KK	3
	Chiang Mai	CM	6
**China**	Guangzhou	GZ	7
	Zhengzhou	ZZ	7
	Beijing	BJ	9
**India**	Banglaore	BA	6
	Kolkata	KO	5
	Trivandrum	TR	4
**Bangladesh**	Dhaka	DH	13
	Mymensingh	MM	17
	Savar	SA	13

**Table 2:** Breakdown of stakeholder participant roles within the livestock industry, by country.

Country	Stakeholder Role			
	Private Industry Leaders	Private Industry Veterinarians	Government Representatives	Agricultural Academics
**China**	15	0	1	9
**Vietnam**	4	3	13	1
**Thailand**	11	4	2	2
**Malaysia**	9	5	5	1
**India**	3	5	1	6
**Bangladesh**	4	2	17	21

**Table 3:** Group ranking of animal welfare issues, by country.

Country	Region	Ranking	
		Farming Context	Slaughter Context
**China**	Beijing	(1) Water (2) Food (3) Temperature (4) Space (5) Shelter (6) Disease (7) Wrong food (8) Rest (9) Fear (10) Boredom (11) Handling	(1) Handling (2) No stunning (3) Fear (4) Temperature (5) Water (6) Wrong food (7) Food (8) Space (9) Rest (10) Boredom (11) Disease (12) Shelter
	Guangzhou	(1) Water (2) Food (3) Shelter (4) Temperature (5) Handling (6) Disease (7) Space (8) Fear (9) Wrong food (10) Rest (11) Boredom	(1) Water (2) Food (3) No stunning (4) Shelter (5) Temperature (6) Handling (7) Disease (8) Space (9) Fear (10) Wrong food (11) Rest (12) Boredom
	Zhengzhou	(1) Food (2) Water (3) Handling (4) Space (5) Temperature (6) Wrong feed (7) Shelter (8) Fear (9) Disease (10) Rest (11) Boredom	(1) No stunning (2) Fear (3) Handling (4) Temperature (5) Food (6) Water (7) Space (8) Wrong food (9) Shelter (10) Rest (11) Disease (12) Boredom
**Vietnam**	Hanoi *	(1a) Food (1b) Water (1c) Wrong food	
		(2a) Fear (2b) No stunning	
		(3a) Rest (3b) Shelter (3c) Temperature	
		(4) Space	
		(5) Handling	
		(6) Disease	
		(7) Boredom	
	Ho Chi Minh *	(1) No stunning	
		(2) Space	
		(3) Disease	
		(4) Handling	
		(5) Temperature	
		(6) Fear	
		(7) Shelter	
		(8) Water	
		(9) Food	
		(10) Rest	
		(11) Wrong food	
		(12) Boredom	
	Ban Me Thout *	(1) No stunning	
		(2) Fear	
		(3) Handling	
		(4) Space	
		(5) Temperature	
		(6) Shelter	
		(7) Rest (8) Water	
		(9) Wrong food	
		(10) Food	
		(11) Disease	
		(12) Boredom	
**Malaysia**	Kuala Lumpur	(1) Space (2) Temperature (3a) Handling (3b) Fear (4a) Water (4b) Food (5) Disease (6) Rest (7) No stunning (8) Boredom	(1) Fear (2) No stunning (3) Temperature (4) Handling (5) Rest (6) Water (7) Space
	Negeri Sembilan	(1) Water	(1) Fear
		(2) Food	(2) Rest
		(3) Shelter	(3) Water
		(4) Temperature	(4) Temperature
		(5) Rest	(5) Shelter
		(6) Wrong food	(6) Handling
		(7) Fear	(7) Space
		(8) Space	(8) No stunning
		(9) Disease	(9) Disease
		(10) Boredom	(10) Boredom
		(11) Handling	(11) Wrong feed
		(12) No stunning	(12) Food
**Thailand**	Bangkok *	(1) Temperature	
		(2) Water	
		(3) Disease	
		(4) Fear	
		(5) Handling	
		(6) Space	
		(7) Food	
		(8) No stunning	
		(9) Rest	
		(10) Shelter	
		(11) Wrong food	
		(12) Boredom	
	Khon Kaen	(1) Water	(1) Handling
		(2) Food	(2) Space
		(3) Wrong food	(3) No stunning
		(4) Space	(4) Rest
		(5) Disease	(5) Shelter
		(6) Handling	(6) Temperature
		(7) Shelter	(7) Fear
		(8) Temperature	(8) Boredom
		(9) Fear	(9) Water
		(10) No stunning	(10) Food
		(11) Rest	(11) Wrong food
		(12) Boredom	(12) Disease
	Chiang Mai	(1) Water	(1) No stunning
		(2) Food	(2) Handling
		(3) Wrong food	(3) Water
		(4) Fear	(4) Fear
		(5) Space	(5) Space
		(6) Disease	(6) Rest
		(7) Handling	(7) Shelter
		(8) Boredom	(8) Temperature
		(9) Rest	(9) Disease
		(10) Temperature	(10) Boredom
		(11) Shelter	(11) Food
		(12) No stunning	(12) Wrong food
**India**	Kolkata *	(1) Disease	
		(2) Food	
		(3) Wrong food	
		(4) Water	
		(5) Space	
		(6) Temperature	
		(7) Rest	
		(8) Shelter	
		(9) Handling	
		(10) No stunning	
		(11) Fear	
		(12) Boredom	
	Bangalore *	(1) No stunning	
		(2) Handling	
		(3) Fear	
		(4) Food	
		(5) Wrong food	
		(6) Shelter	
		(7) Shelter	
		(8) Disease	
		(9) Rest	
		(10) Space	
		(11) Temperature	
		(12) Boredom	
	Trivandrum *	(1) Food	
		(2) Water	
		(3) Wrong food	
		(4) Shelter	
		(5) Space	
		(6) Temperature	
		(7) No stunning	
		(8) Disease	
		(9) Fear	
		(10) Rest	
		(11) Handling	
		(12) Boredom	
**Bangladesh**	Dhaka	(1) Space (2) Rest (3) Wrong food (4) Fear (5) Handling (6) *Transportation discomfort* (added by participants) (7) Temperature (8) Boredom (9) Shelter (10) Disease (11) Water quality (added by participants) (12) Food (13) Water (14) No stunning	(1) *Transportation discomfort* (added by participants) (2) Fear (3) Handling (4) Slaughtering pregnant animals unknowingly (added by participants) (5) Disease (6) Rest (7) No stunning
	Mymensingh	(1) Wrong food	(1) Handling
		(2) Space	(2) Shelter
		(3) Food	(3) Rest
		(4) Shelter	(4) Water
		(5) Disease	(5) Temperature
		(6) Handling	(6) Food
		(7) Temperature	(7) Wrong food
		(8) Rest	(8) Disease
		(9) Fear	(9) Fear
		(10) Water	(10) Space
		(11) No stunning	(11) No stunning
		(12) Boredom	(12) Boredom
	Savar	(1) Food	(1) *Poor vehicle design* (added by participants)
		(2) Wrong food	(2) Rest
		(3) Disease	(3) Temperature
		(4) Temperature	(4) Space
		(5) Space	(5) Handling
		(6) Poor vehicle design (added by participants)	(6) Shelter
		(7) Handling	(7) Water
		(8) Rest	(8) Fear
		(9) Shelter	(9) Disease
		(10) Fear	(10) Food
		(11) Water	(11) Boredom
		(12) Boredom	(12) No stunning

* Farming and slaughter combined; Animal Welfare Issues as Stated on Activity Cards in Full (Translated): Disease—Lack of treatment of disease and injury; Food—Not enough food; Wrong food—Inappropriate feed; Water—Not enough water; Space—Confinement in space too small to express normal behaviour; Temperature—Thermal discomfort: too hot or cold; Rest—Lack of comfortable rest; Shelter—Lack of shelter; Handling—Rough stock handling; No stunning—Lack of stunning during slaughter; Fear—Experiencing fear and distress; Boredom—Boredom.

**Table 4:** Solutions to improving animal welfare presented by livestock leaders in each location.

	China			Vietnam			Thailand			Malaysia		India			Bangladesh		
	Beijing	Guangzhou	Zhengzhou	Hanoi	Ho Chi Minh City	Ban Me Thout	Bangkok	Chiang Mai	Khon Kaen	Kuala Lumpur	Negeri Sembilan	Kolkata	Bangalore	Trivandrum	Dhaka	Mymensingh	Savar
Childhood education				X	X	X	X			X	X	X	X	X	X		
Industry training				X	X	X	X	X	X	X		X		X	X	X	X
Build public awareness		X	X	X	X		X			X	X	X		X	X		X
Build AW profile using mass media	X		X		X			X				X	X		X		X
Build AW profile using social media	X		X					X	X			X	X	X	X		
Societal move to larger and licensed slaughterhouses for ease of monitoring						X					X			X			
Local research on issues and local holistic solutions	X	X											X	X		X	
Engage cultural pride/goodness in heritage								X	X			X	X	X			
Create prescriptive and consistent standards and company policy	X	X	X	X					X		X	X			X	X	X
Focus on science to eliminated emotive stigma												X	X				
Focus on One Welfare, and the holistic human tie												X		X	X	X	X
Focus on business benefits to improving animal welfare	X	X	X	X	X			X		X	X					X	X
Consumer willingness to pay campaigns	X						X										
Collaborations between stakeholder groups (industry, industry groups, NGO, government, universities)		X	X	X			X	X	X	X	X	X			X		
Regional flexibility and understanding for standards							X										
Peer skill sharing								X	X	X							
Leverage food safety or antimicrobial resistance for AW			X							X					X		X
Leverage new technology to safeguard animal welfare			X														
Incorporate animal welfare into religious curriculum										X				X		X	

**Table 5:** Willingness to learn calmer animal handling techniques, from a scale of 1–10 (not willing—extremely willing), by location.

	*n*	Mean *	Median *
**Bangladesh**			
Dhaka	9	9.7	10
Mymensingh	8	6	5.5
Savar	11	9.4	10
**China**			
Beijing	9	8	7.5
Guangzhou	7	8.4	8
Zhengzhou	7	6	6
**India**			
Kolkata	5	7.6	8
Bangalore	6	7	7.5
Trivandrum	4	4.4	4
**Malaysia**			
Kuala Lumpur	12	7.6	8
Negeri Sembilan	5	1.6	2
**Thailand**			
Bangkok	9	7.5	7
Chiang Mai	6	9	10
Khon Kaen	3	8.3	8
**Vietnam**			
Hanoi	5	7.5	7.5
Ban Me Thout	5	6.2	6
Ho Chi Minh City	5	7	7

* 1 being stakeholders who are extremely unlikely to embrace stunning, 10 being extremely likely.

**Table 6:** Evidence supported opportunities for international animal welfare initiatives operating in Asia.

**China**	Development of prescriptive standards based on science and economic modelling Support the development of local farm animal welfare research Clearly communicate the business benefits for improving farm animal welfare in industry forums Continue building the profile of animal welfare amongst the general public on Chinese social media platforms such as Weibo, Huajiao, and Wechat, including food blog messages of improved product quality and taste Create documentaries (partnering with state media and government) on farm animal welfare
**India**	Develop school-based education on animal empathy, utilising the cultural history of reverence for animals; including Indian heritage stories of the symbiotic relationship between humans and animals to support a reconnection to both Indian cultural heritage and animals Support local research to holistically understand animal welfare issues in detail that will enable the development of tailored strategies and issue-specific programs that will also benefit stakeholder livelihoods Raise the awareness of the general public to animal welfare by hosting social media campaigns that feature celebrities, and leverage the Hindu and Indian cultural heritage of reverence for animals. Popular platforms locally include YouTube and WhatsApp Tie animal welfare to televisual public announcement campaigns that support the concept of One Welfare, featuring human welfare-related issues, such as rabies management Ensure regulatory bodies, such as the Animal Welfare Board of India, are inclusive of stakeholders that represent communities responsible for animal welfare at critical points in their lives, such as the inclusion of Muslim representatives for the livestock processing community
**Thailand**	Develop industry stakeholder animal welfare training that focusses on how to improve animal welfare, which is best coordinated by the Department of Livestock Development in collaboration with livestock associations, with non-government organisation coordination assistance if requested Facilitate peer sharing events and platforms based on animal welfare, that involve livestock association groups and utilise knowledgeable farmers to share experiences and advice Leverage the local culture of kindness and empathy to build education programs and initiatives that are backed with animal welfare knowledge Advocate childhood and community education based on existing empathy and Buddhist ideals to improve animal welfare in locations such as community halls and temples Produce clear and consistent animal welfare standards that are tailored to the Thai environment, and advocate international acceptance of these standards for export
**Vietnam**	Develop industry training on what animal welfare is, why animal welfare should be addressed and what the benefits are. Follow-up training to be offered on how to deliver improved animal welfare. Both would be best hosted by livestock associations or international business partners, in collaboration with the relevant government agency Research the perception of children to animals in Vietnam and develop a childhood education program that fosters empathy and concern for animals at school
**Malaysia**	Develop collaborative educational programs to be hosted by the Department of Veterinary Services (DVS) to build the capacity and knowledge of JAKIM (the Malaysian Islamic Authority) on farm animal welfare, to increase understanding around pre-slaughter stunning and other matters of animal welfare Develop technical training programs to be hosted by DVS for industry, to build awareness of business benefits for improving animal welfare, and best practice standards Advocate the addition of agricultural education for children to focus on where their food comes from, and why it is important to care Develop campaigns that draw on the religious mandate outlined in Islamic texts to be careful guardians of animal welfare Develop public awareness campaigns that tie improved welfare products to quality
**Bangladesh**	Support the development of internationally-advised, locally-devised standards for livestock welfare Create a network of representatives interested in progressing the field of animal welfare in livestock production in Bangladesh, including academics, government veterinarians and key industry stakeholders Collaborate with the network of representatives to create animal welfare training materials for government representatives, and for farmers, that focus on the human welfare benefits for improving animal welfare, and the fundamentals to improving mutual welfare (in a One Welfare framework) Deliver animal welfare education by training trainers, thus empowering the growth of animal welfare specialists within Bangladesh Develop a smart phone application on which the training materials are freely available. This application could also host the animal welfare network and be advertised on social media Develop public awareness campaigns that tie farm animal welfare to food safety and antibiotic resistance Produce a children’s show in the likeness of a popular existing Bangladeshi show that encourages the empowerment of women and that holistically focusses on animal welfare and human welfare

## Appendix A

**Table A1:** Focus group structure and base questions.

Question Number	Question	Q Category (Analysis Intentions)	Approx. Time Allocation
1	Please introduce yourselves by stating your name and where you work.	Introductory (not used in analysis)	10 min
2	What are the benefits you see to improving animal welfare?	Transition	10 min
3	Share top 5 modes of encouragement (write on board, or flipchart). In recent research we completed, stakeholders in your country said they were more encouraged to improve animal welfare if the changes were prescribed in some specific ways. These are the top 5 ways. Can you please share your thoughts and ideas on the reasons why the stakeholders rated this so highly in this country?	Key (all comments included in thematic analysis)	15 min
4	Follow on from last question. What might be the best strategies to use this information to motivate stakeholders to improve animal welfare?	Key (all comments included in thematic analysis)	10 min
5	Share top 5 factors’ impacting ability (write on board, or flipchart). In recent research we completed, stakeholders in your country rated the following as the top 5 factors that impact their ability to improve animal welfare during slaughter and transport. Can you please share your thoughts and ideas on the reasons the stakeholders rated this so highly in this country?	Key (all comments included in thematic analysis)	15 min
6	Follow on from last question. What might be the best ways to support stakeholders to make improvements to animal welfare?	Key (all comments included in thematic analysis)	10 min
	Coffee break + prepare cards for Q3 follow on		15 min
7	Group activity (ranking exercise). As a group, please place these cards in rank, from the greatest animal welfare concern in this country to the least. Provide the group cards with listed animal welfare concerns during slaughter and transport.	Key (record ranking for analysis, transcribe key comments during activity for analysis)	10 min
8	Individual activity. Please write down 3 changes that could be made in your workplace to improve animal welfare. Please each share the most important one.	Key (record response for analysis)	10 min
9	Follow on from Q3. Ranking activity. Based on your comments at the start, we made cards of the benefits for improving animal welfare. As a group, can you please place them in order of most important to least important.	Key (record ranking for analysis, transcribe key comments during activity for analysis)	10 min
10	On a scale of 1-10, 1 being extremely unlikely and 10 being extremely likely, how willing are stakeholders to embrace stunning prior to slaughter to ensure the animal is completely unconscious before killing it? Move around the table one by one.	Transition (record rating for analysis, transcribe key comments during activity for analysis)	10 min
11	How could stunning be implemented where it isn’t already?	Key (all comments included in thematic analysis)	10 min
12	On a scale of 1-10, 1 being extremely unlikely and 10 being extremely likely, how willing are stakeholders to improve their stockpersonship skills to be calmer with the animals?	Transition (record rating for analysis, transcribe key comments during activity for analysis)	10 min
13	How could stakeholders be encouraged to improve their stockpersonship skills?	Key (all comments included in thematic analysis)	10 min
14	Of all of the points you have shared with us today, what is the most important?	Key (all comments included in thematic analysis)	15 min
15	Present a short oral summary of the key points as presented by the participants. Does that capture the most important points of today? Have we missed anything?	Closing	5 min

**Note:** the data and analysis resulting from the shaded questions are included within this manuscript. Data resulting from all other questions is presented within publications addressing each subject separately.

## References

[1] McQuarrie E. (2011). The Market Research Toolbox: A Concise Guide for Beginners.

[2] Andreasen A.R., Kotler P. (2008). Strategic Marketing for Nonprofit Organizations.

[3] Entwistle N., Ramsden P. (2015). Understanding Student Learning (Routledge Revivals).

[4] Berry J. (2005). Nonprofits and civic engagement. Public Adm. Rev..

[5] Evans M. (2013). The Importance of Really Knowing Your Target Audiences. http://www.forbes.com/sites/markevans/2013/03/20/the-importance-of-really-knowing-your-target-audiences/-411c27ce6ddc.

[6] Verbeke W. (2009). Stakeholder, citizen and consumer interests in farm animal welfare. Anim. Welf..

[7] Kauppinen T., Vainio A., Valros A., Rita H., Vesala K.M. (2010). Improving animal welfare: Qualitative and quantitative methodology in the study of farmers’ attitudes. Anim. Welf..

[8] Vanhonacker F., Verbeke W., Van Poucke E., Tuyttens F.A.M. (2008). Do citizens and farmers interpret the concept of farm animal welfare differently?. Livest. Sci..

[9] Bock B.B., Van Huik M.M. (2007). Animal welfare: The attitudes and behaviour of European pig farmers. Br. Food J..

[10] Sinclair M., Idrus Z., Yan W., van Nhiem D., Na_Lampang P., Zito S., Phillips C.J.C. (2017). Attitudes of stakeholders to animal welfare during slaughter and transport in SE and E Asia. Anim. Welf..

[11] Li X., Zito S., Sinclair M., Phillips C.J.C. (2018). Perceptions of animal welfare issues during Chinese transport and slaughter of livestock by a sample of stakeholders in the industry. PLoS ONE.

[12] Sinclair M., Zito S., Phillips C.J.C. (2017). The Impact of stakeholders’ roles within the livestock industry on their attitudes to livestock welfare in Southeast and East Asia. Animals.

[13] Sinclair M., Morton J., Phillips C.J.C. (2018). Turning Intentions into Animal Welfare Improvement in the ASIAN Livestock Sector. J. Appl. Anim. Welf. Sci..

[14] Gryzbowski A., McCaffrey S.C., Paisley K.R. (2010). Beyond International Water Law: Successfully Negotiating Mutual Gains Agreements for International Watercourses. University of the Pacific Scholarly Commons. McGeorge Glob. Busi. Dev..

[15] Mayers J., Vermeulen S. (2002). Company-Community Forestry Partnerships: From Raw Deals to Mutual Gains.

[16] Sinclair M., Phillips C.J.C. (2018). Key tenets of operational success in international animal welfare initiatives. Animals.

[17] Sinclair M., Phillips C. (2019). Percived benefits to improving animal wefare from the cross-cultural perspective of Asian industry stakeholders. Animals.

[18] Vaismoradi M., Jones J., Turunen H., Snelgrove S. (2016). Theme development in qualitative content analysis and thematic analysis. J. Nurs. Educ. Pract..

[19] Maslow A. (1943). A theory of human motivation. Psychol. Rev..

[20] Wynne C.D.L. (2004). The perils of anthropomorphism. Nature.

[21] Burns G.L., Burns G.L., Paterson M. (2014). Anthropomorphism and Animals in the Anthropocene. Engaging with Animals: Interpretations of a Shared Experience.

[22] Bekoff M. (2007). The Emotional Lives of Animals.

[23] Ahsan M., Hasan B., Algotsson M., Sarenbo S. (2014). Handling and welfare of bovine livestock at local abattoirs in Bangladesh. J. Appl. Anim. Welf. Sci..

[24] Federation of Veterinarian of Europe (FVE) (2002). Slaughter without Stunning and Food Labeling: Briefing Note. https://www.veterinaire.fr/fileadmin/user_upload/documents/outils-et-services/Index_juridique/Premiere_lettre_de_M_a_Z/Religious_slaughter_and_food_chain.pdf.

[25] Gibson T.J., Johnson C.B., Murrell J.C., Hulls C.M., Mitchinson S.L., Stafford K.J., Johnstone A.C., Mellor D.J. (2009). Electroencephalographic responses of halothane-anaesthetised calves to slaughter by ventral-neck incision without prior stunning. N. Z. Vet. J..

[26] Gregory N.G., Fielding H.R., von Wenzlawowicz M., von Holleben K. (2010). Time to collapse following slaughter without stunning in cattle. Meat Sci..

[27] Phillips C., Izmirli J.C., Aldavood S., Alonso S.J., Choe M., Hanlon B.I., Handziska A., Illman A.G., Keeling L., Kennedy M. (2012). Students’ attitudes to animal welfare and rights in Europe and Asia. Anim. Welf..

[28] Ling R., Zulkifli I., Lampang P.N., Nhiem D.V., Wang Y., Phillips C.J.C. (2016). Attitudes of students from south-east and east Asian countries to slaughter and transport of livestock. Anim. Welf..

[29] Sinclair M., Phillips C. (2017). The cross-cultural importance of animal protection and other world social issues. J. Agric. Environ. Ethics.

[30] Hofstede Insights (2019). Country Comparison. https://www.hofstede-insights.com/country-comparison/china,malaysia,thailand,vietnam/.

[31] PEW Research Centre Forum on Religion and Public Life and Global Religious Landscape. 18 December 2012. https://www.pewforum.org/2012/12/18/global-religious-landscape-exec/.

[32] Office of the Registrar General and Census Commissioner India (2001). Distribution of Population by Religion. http://censusindia.gov.in/Census_And_You/religion.aspx.

[33] Pinillos R.G., Appleby M.C., Manteca X., Scott-Park F., Smith C., Velarde A. (2016). One welfare—A platform for improving human and animal welfare. Vet. Rec..

[34] World Bank (2016). Poverty and Equity Data Portal. http://povertydata.worldbank.org/.

[35] The Economist Intelligence Unit (2019). Global Access to Healthcare Index. http://accesstohealthcare.eiu.com/.

[36] United Nations Development Programme (2014). Human Development Data. http://hdr.undp.org/en/data.

[37] Sima Y., O’Sullivan S. (2016). Chinese animal protection laws and the globalization of welfare norms. Int. J. Law Context.

[38] Act of the Parliament of India (1960). The Prevention of Cruelty to Animals Act. https://indiacode.nic.in/bitstream/123456789/1547/1/196059.pdf.

[39] Anon (2018). What Is the Punishment for Animal Abuse in Bangladesh?. https://www.dhakatribune.com/bangladesh/law-rights/2018/02/28/punishment-animal-abuse-bangladesh.

[40] Laws of Malaysia Act 772 (2015). Animal Welfare Act. https://www.aaalac.org/resources/Malaysia.pdf.

[41] Prevention of Cruelty and Animal Welfare Provision Act (2016). National Legislative Assembly of Thailand. http://app-thca.krisdika.go.th/Naturesig/CheckSig?whichLaw=law2&folderName=%BB53&lawPath=%BB53-20-2557-a0001.

[42] Anon New Vietnam Law Mandates Humane Treatment of Farm Animals. https://blog.humanesociety.org/2018/11/new-vietnam-law-mandates-humane-treatment-of-farm-animals.html.

